# Distinct immunologic kinetics and cytomegalovirus reactivation incidence with rituximab- versus obinutuzumab–bendamustine in follicular lymphoma: a single-center case series study

**DOI:** 10.1007/s00432-025-06351-2

**Published:** 2025-11-01

**Authors:** Matteo D’Addona, Luca Pezzullo, Lorenzo Settembre, Emilia Vaccaro, Roberto Guariglia, Bianca Serio, Laura Mettivier, Andrea Gigantiello, Giovanni Signorile, Angela Bertolini, Francesca Picone, Bianca Cuffa, Valentina Giudice, Carmine Selleri

**Affiliations:** 1 Department of Medicine, Surgery and Dentistry “Scuola Medica Salernitana”, University of Salerno, 84081 Baronissi, Italy; 2 Hematology and Transplant Center, University Hospital “San Giovanni di Dio e Ruggi d’Aragona”, Salerno, Italy; 3 Molecular Biology Section, University Hospital “San Giovanni di Dio e Ruggi d’Aragona”, 84131 Salerno, Italy

**Keywords:** Cytomegalovirus, Follicular lymphomas, Immune reconstitution, Viral reactivation, Anti-CD20 monoclonal antibodies

## Abstract

Rituximab and obinutuzumab, anti-CD20 monoclonal antibodies, are widely employed for treatment of follicular lymphoma (FL) by targeting both neoplastic and normal CD20-expressing B lymphocytes, consequently inducing immunosuppression. In this condition, viral reactivations are common and represent a major cause of morbidity and mortality. In this single-center two-arm observational real-life study, we evaluated incidence, immunological and serological status, and clinical outcomes of cytomegalovirus (CMV) reactivation in FL patients treated with bendamustine + rituximab (R-BENDA; *N* = 23) or obinutuzumab (G-BENDA; *N* = 23). CMV reactivation more frequently occurred in patients treated with R-BENDA compared to G-BENDA (*P* = 0.022), and immune kinetics showed significant differences between the two groups, with a deeper and earlier immunosuppression in R-BENDA treated subjects. Moreover, R-BENDA group displayed a significant higher risk of CMV reactivation compared to G-BENDA (hazard ratio, 2.232; 95% confidence interval 1.107–4.500; *P* = 0.0249). However, G-BENDA patients tended to have a shorter 5-year overall survival (59% vs. 86.7%, G-BENDA vs. R-BENDA; *P* = 0.0903). By multivariate analysis, B symptoms were an independent predictor of CMV reactivation, and high baseline SUV as an additional risk factor in R-BENDA patients. In conclusion, anti-CD20 agent could increase the risk of CMV reactivation in FL patients, especially in R-BENDA treated subjects who experienced early and deep immunosuppression. Therefore, close monitoring of clinical and laboratory data may improve outcomes in FL patients by preventing CMV disease, especially in those treated with rituximab who are more prone to viral reactivation. However, larger prospective studies are required to confirm our preliminary results.

## Introduction

Follicular lymphoma (FL), an indolent, yet incurable, non-Hodgkin lymphoma (NHL), arises from follicular B cells and is characterized by slow progression, with patients often experiencing relapses over time (Qualls and Salles 2022). FL is the second most common subtype of NHL, accounting for approximately 20–30% of all cases of all lymphomas worldwide, and primarily affects adults, with a median age at diagnosis of 60–65 years (Qualls and Salles 2022; Weiner 2010). Its incidence has been increasing over the past few decades, likely due to improved detection methods and an aging population (Weiner 2010). First-line therapy for FL typically includes anti-CD20 monoclonal antibodies (moAbs), such as rituximab and obinutuzumab, combined with standard chemotherapy. The introduction of anti-CD20 monoclonal antibodies has dramatically modified clinical outcomes of these patients, making FL a chronic disease (Herting et al. 2014).

Rituximab, the first-in-class chimeric moAb targeting CD20, recognizes a cell surface protein expressed on both neoplastic and mature B lymphocytes, and induces B cell depletion through antibody-dependent cellular cytotoxicity (ADCC), complement-dependent cytotoxicity (CDC), and apoptosis (Pavlasova and Mraz 2020). Rituximab is widely used for treatment of FL, as monotherapy or in combination with chemotherapy regimens, including CHOP or bendamustine, showing longer progression-free survival (PFS) and overall survival (OS) (Qualls and Salles 2022; Fowler 2011; Chan et al. 2024). Obinutuzumab is a humanized, glycoengineered anti-CD20 moAb, and differs from rituximab in its enhanced ADCC action and increased binding affinity to CD20. Obinutuzumab has shown a potential superior efficacy compared to rituximab and is increasingly used in the treatment of FL, particularly in combination with bendamustine. In the GALLIUM clinical trial, obinutuzumab has improved PFS of FL patients with higher rates of complete response (CR) (Hiddemann et al. 2018; Townsend et al. 2023; Flinn et al. 2019). While generally well tolerated, anti-CD20 moAbs are associated with several toxicities, including infusion-related reactions, which are the most common, and immunosuppression, increasing the risk of infectious complications (Park and Kim 2024). Bendamustine, a chemotherapy agent with both alkylating and purine analog properties, enhances the effects of anti-CD20 antibodies, leading to prolonged lymphopenia, especially CD4 + depletion, raising susceptibility to opportunistic infections (Lalic et al. 2022; Fung et al. 2019; Saito et al. 2015; Isono et al. 2016; Saburi et al. 2023; Pezzullo et al. 2021).

Cytomegalovirus (CMV), a human beta herpesvirus, has a seroprevalence of ~ 83% in the general population, and clinical presentation varies from asymptomatic in healthy individuals to severe organ dysfunction in immunocompromised patients, such as those with hematological disorders or solid organ or hematopoietic stem cell transplant recipients (Serio et al. 2021). After primary infection, immunocompetent individuals typically remain lifelong silent carriers, unless specific conditions, such as immunosuppression, which leads to viral reactivation. The risk of CMV reactivation is heightened in patients undergoing treatment with anti-CD20 moAbs, because of their B lymphocyte depletion, causing hypogammaglobulinemia and reduced T cell activation (Karaman et al. 2025). CMV reactivation in immunocompromised subjects can lead to a severe disease, with hepatitis, severe pneumonia, nervous system manifestations, myelosuppression, and graft rejection in transplant recipients (Azevedo et al. 2015; Ong et al. 2022). Indeed, CMV reactivation remains one of the leading causes of morbidity and mortality in hematological patients and in solid organ transplant recipients (Serio et al. 2021). Therefore, vigilant monitoring of CMV status is crucial in hematological patients to manage and prevent potentially life-threatening infections, and specific international recommendations are outlined by the ECIL consensus and other working groups (Ljungman et al. 2019, 2025; Girmenia et al. 2025; Piñana et al. 2024).

Despite well-established efficacy and safety profiles of anti-CD20 moAbs in NHL treatment, comparative real-world data on CMV reactivation incidence, outcomes, and immune profiles in FL patients treated with R-BENDA or G-BENDA remain limited. In this single-center two-arm observational real-life study, we evaluated immunological kinetics, clinical outcomes, and predictors of CMV reactivation in FL patients treated with immunochemotherapy regimens containing bendamustine + rituximab or obinutuzumab in a real-life setting.

## Patients and methods

### Patients

A total of 46 consecutive patients diagnosed with FL at the Hematology Unit, University Hospital “San Giovanni di Dio e Ruggi d’Aragona”, Salerno, Italy, from April 2013 to December 2024, were included in this single-center real-life retrospective observational exploratory study. Clinical characteristics at enrollment are summarized in Table 1. FL diagnosis was made according to World Health Organization (WHO) criteria (Alaggio et al. 2022), and chemotherapy administered as per international guidelines (Jacobsen 2022). Inclusion criteria were: age ≥ 18 years old; diagnosis of FL; positive CMV serological status at diagnosis; and presence of informed consent. Exclusion criteria were: age < 18 years old; diagnosis of other NHL by histology examination; negative CMV serological status at diagnosis; and lack of informed consent. This study was conducted in accordance with the Declaration of Helsinki and protocols approved by our local Ethics Committee ‘‘Campania Sud’’ (Brusciano, Naples, Italy; prot./SCCE n. 24,988). Patients received bendamustine in combination with rituximab or obinutuzumab, and was given at doses of 70–90 mg/m^2^ for two consecutive days every 21 days for four to six cycles, while rituximab was administered at 375 mg/m^2^ every 21 days and obinutuzumab at 1000 mg the first day of every cycle.

**Table 1:** Clinical characteristics at baseline

Characteristic	Entire cohort *N* = 46	*R*-Bendamustine *N* = 23	G-Bendamustine *N* = 23	*P* value
Mean age, years (range)	64 (40–86)	66 (51–86)	61 (40–78)	0.0919
M/F	24/22	11/12	12/11	> 0.9999
Grade				0.4552
1	11	5	6	
2	19	8	11	
3a	13	8	5	
3b	2	2	0	
N.E.	1	–	1	
Stage, n				0.5963
I	1	1	0	
II	6	4	2	
III	15	6	9	
IV	24	12	12	
FLIPI, n				0.1581
Low risk	8	6	2	
Intermediate risk	17	6	11	
High risk	23	13	10	
Symptoms, n				> 0.9999
A	40	20	20	
B	6	3	3	
Mean nodal involvement, n (range)	5 (1–23)	5 (1–12)	9 (2–23)	0.0310
Mean SUV (range)	8.3 (2–23)	7.4 (3.1–21.5)	9.6 (2.0–27.0)	0.1695
Mean Hb, g/dL (range)	13.5 (10.2–16.4)	13.9 (10.8–16.3)	13 (7.8–16.4)	0.0809
Mean platelets/µL (range)	199,119 (66,000–352,000)	198,800 (117,000–352,000)	203,348 (66,000–342,000)	0.8182
Mean LDH, (range)	433.5 (166–1102)	482 (166–1102)	387 (201–718)	0.0813
Mean albumin, g/dL (range)	4.2 (3.2–5)	4.2 (3.5–5.0)	4.1 (2.3–4.9)	0.6978
Bendamustine dosage				0.4591
70	9	6	3	
90	37	17	20	
Median cycles, n (range)	6 (2–6)	6 (3–8)	6 (2–6)	0.9143
CMV serology				0.6652
Positive	40 6	21 2	19 4	
Negative				0.0220
CMV reactivation, n (%)	37 (80)	22 (96)	15 (65)	

### Flow cytometry

For flow cytometry immunophenotyping, as part of routine clinical practice, fresh EDTA whole peripheral blood was obtained at enrollment, at each cycle, and at + 6, +9, and + 12 months post-treatment. Briefly, samples were stained with the following antibodies: CD45; CD3; CD4; and CD8, as per manufacturer’s instructions (Beckman Coulter, Brea, CA, USA), incubated for 20 min in the dark at room temperature, red blood cell lysed, washed with 2mL of phosphate buffer saline (PBS; Beckman Coulter), and then resuspended in 500 µL of PBS for acquisition. Specimens were acquired using a five-color FC500 cell analyzer cytometer (Beckman Coulter, ), or on a ten-color three-laser Navios EX or DxFlex cytometers (Beckman Coulter). At least 1 million events per sample were recorded. Post-acquisition analysis was performed using CPX, Navios tetra, or Kaluza C software (Beckman Coulter). First, debris were removed, and lymphocytes were identified using linear parameters (forward scatter area vs. side scatter area). Next, CD3 expression was studied, and on CD3 + T cells, CD4 and CD8 expression was further investigated.

### CMV quantification and serology

Immunoglobulin (Ig) levels (IgG, IgM, and IgA) were measured at each time point, as part of routine clinical practice, and hypogammaglobulinemia was defined as total IgG levels < 400 mg/dL. Plasma CMV-DNA copy number was quantified by real-time TaqMan CMV-DNA PCR according to manufacturers' instructions (Roche), and at each time point. After diagnosis of CMV reactivation, CMV-DNA levels were assessed weekly until clearance. The instrument cut-off for positive results was CMV-DNA copy number > 137 copies/μL, as previously described (Pezzullo et al. 2021). CMV reactivation was defined as CMV-DNA copy number > 137 copies/μL in anti-CMV IgG + patients without organ damage, while CMV disease was defined when CMV-related organ damage occurred, including pneumonia, gastrointestinal disease, hepatitis, retinitis, central nervous system disease, nephritis, myocarditis, and pancreatitis, associated to the detection of CMV-DNA on the involved organ. Anti-CMV agents were started when progressively increasing weekly in CMV-DNA levels were observed. We also distinguished between asymptomatic CMV reactivation and CMV disease with organ involvement. Reactivation was managed with weekly monitoring and pre-emptive therapy (valganciclovir or intravenous ganciclovir) in case of progressive viremia.

### Statistical analysis

Data were collected in spreadsheets and were analyzed using R statistical software (v. 4.0.5; RStudio) and SPSS (v. 25; IBM). Continuous variables were expressed as mean ± SD or median with range where appropriate. Differences between groups were assessed by Chi-square, Fisher’s, Wilcoxon signed‐rank, or unpaired two‐tailed *t* tests. Kaplan–Meyer, log‐rank, and Breslow tests were used for survival analysis, and univariate and multivariate Cox regression models were used to examine effects (odd ratio, OR) of independent variables on outcomes. All statistical tests were two-sided, and a *p* value < 0.05 was considered statistically significant.

## Results

### Clinical characteristics at baseline

Among enrolled patients, 23 of them (50%) received R-BENDA (mean age, 66 years old; range 51–86 years; M/F, 11/12) and 23 G-BENDA (mean age, 61 years old; range 40–78 years; M/F, 12/11), with similar demographics distributions between groups (Table 1). Patients treated with G-BENDA showed a predominance of grade 2 FL (*N* = 11; 48%), while patients treated with R-BENDA showed a lower number of nodal sites involved compared to G-BENDA (5 vs. 9 nodal sites, *P* = 0.0310), as well as a slightly lower mean maximum standardized uptake value (SUV) by PET scan (mean SUV, 7.4 vs. 9.6, R-BENDA vs. G-BENDA; *P* = 0.1695), thus indicating a higher tumor burden at baseline in the obinutuzumab-treated cohort. Baseline complete blood counts and biochemistry parameters were comparable between groups. Bendamustine was administered at 70 mg/m^2^ in 6 patients (26%) and 90 mg/m^2^ in 17 patients (74%) in the R-BENDA arm, and in 3 (13%) and 20 (87%) patients, respectively, in the G-BENDA group. The median number of cycles was 6 in both groups (range 3–8 for R-BENDA; and 2–6 for G-BENDA; *P* = 0.9143).

### Immunological kinetics

**Fig. 1:**
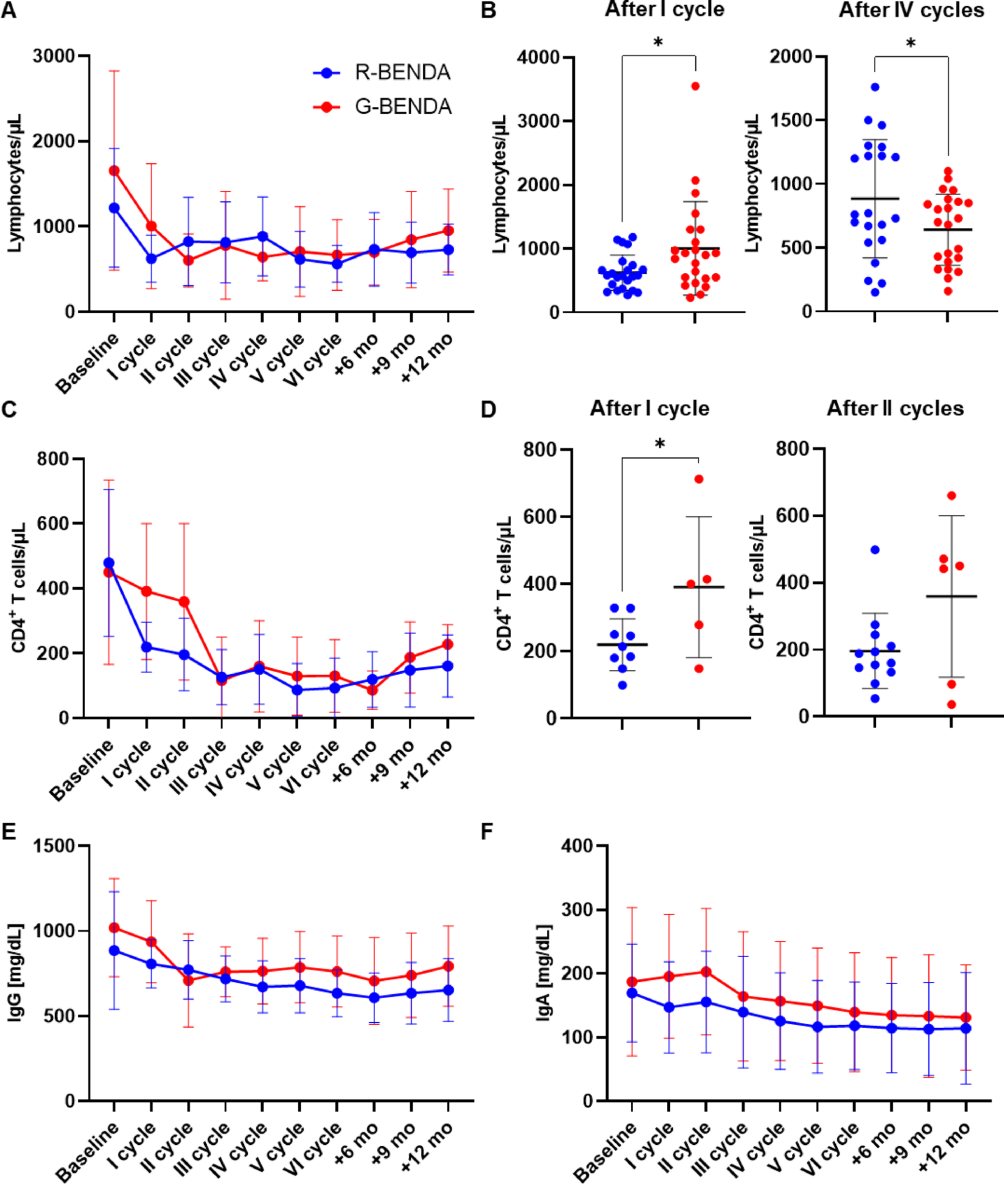
Immunological kinetics during rituximab + (R-BENDA) or obinutuzumab + bendamustine (G-BENDA) treatment in follicular lymphoma patients. **A** Longitudinal analysis of lymphocyte counts at baseline, during treatment cycles, and at follow-up (+ 6, + 9, and + 12 months) in patients treated with R-BENDA or G-BENDA. **B** Scatter plots showing individual lymphocyte counts at first and fourth cycle. **C** Longitudinal analysis of CD4 + T cell counts at baseline, during treatment cycles, and at follow-up (+ 6, + 9, and + 12 months) in patients treated with R-BENDA or G-BENDA. **D** Scatter plots of individual CD4 + T cell counts after the first and second cycle. Longitudinal analysis of **E** serum immunoglobulin (Ig) G and **F** serum IgA levels at baseline, during treatment cycles, and at follow-up (+ 6, + 9, and + 12 months) in patients treated with R-BENDA or G-BENDA. Data are shown as mean ± SD. *P < 0.05

Next, longitudinal immunological kinetics was investigated in patients treated with R-BENDA and compared to those receiving G-BENDA (Fig. 1A). Following treatment initiation, R-BENDA induced a rapid and profound lymphocytopenia after the first cycle (mean ± SD, 624 ± 275 vs. 1005 ± 734 cells/µL, R-BENDA vs. G-BENDA; *P* = 0.0272) (Fig. 1B), conversely, G-BENDA caused a delayed but deeper lymphocytopenia after four cycles of therapy (mean ± SD, 884 ± 463 vs. 642 ± 279 cells/µL, R-BENDA vs. G-BENDA; *P* = 0.0405). However, total absolute lymphocyte counts remained low throughout therapy and follow-up, without a complete recovery in both groups.

We next focused on CD4 + T-lymphocytes, given their critical role in adaptive immunity (Fig. 1C). Of note, R-BENDA regimen induced a more rapid, pronounced, and prolonged CD4 + T cell reduction compared to G-BENDA, starting from the first cycle (mean ± SD, 220 ± 77 vs. 391 ± 210 cells/µL, R-BENDA vs. G-BENDA; *P* = 0.0444) (Fig. 1D). Similar to that report for total lymphocytes, absolute CD4 + T cell counts remained below baseline levels throughout therapy and follow-up, without a complete recovery in both groups.

Subsequently, we also studied humoral immunity by measuring serum immunoglobulin (Ig) G and IgA levels. R-BENDA regimen induced a constant progressive decline in circulating IgG, reaching the nadir at the end of therapy (mean ± SD, 634 ± 138 vs. 762 ± 209 cells/µL, R-BENDA vs. G-BENDA; *P* = 0.0473) and remaining lower at 12-month follow-up (mean ± SD, 653 ± 185 vs. 794 ± 236 cells/µL, R-BENDA vs. G-BENDA; *P* = 0.0707) (Fig. 1E). For IgA serum levels, both regimens induced a progressive decline in their concentrations, which remained below normal ranges also during follow-up (Fig. 1F).

**Fig. 2:**
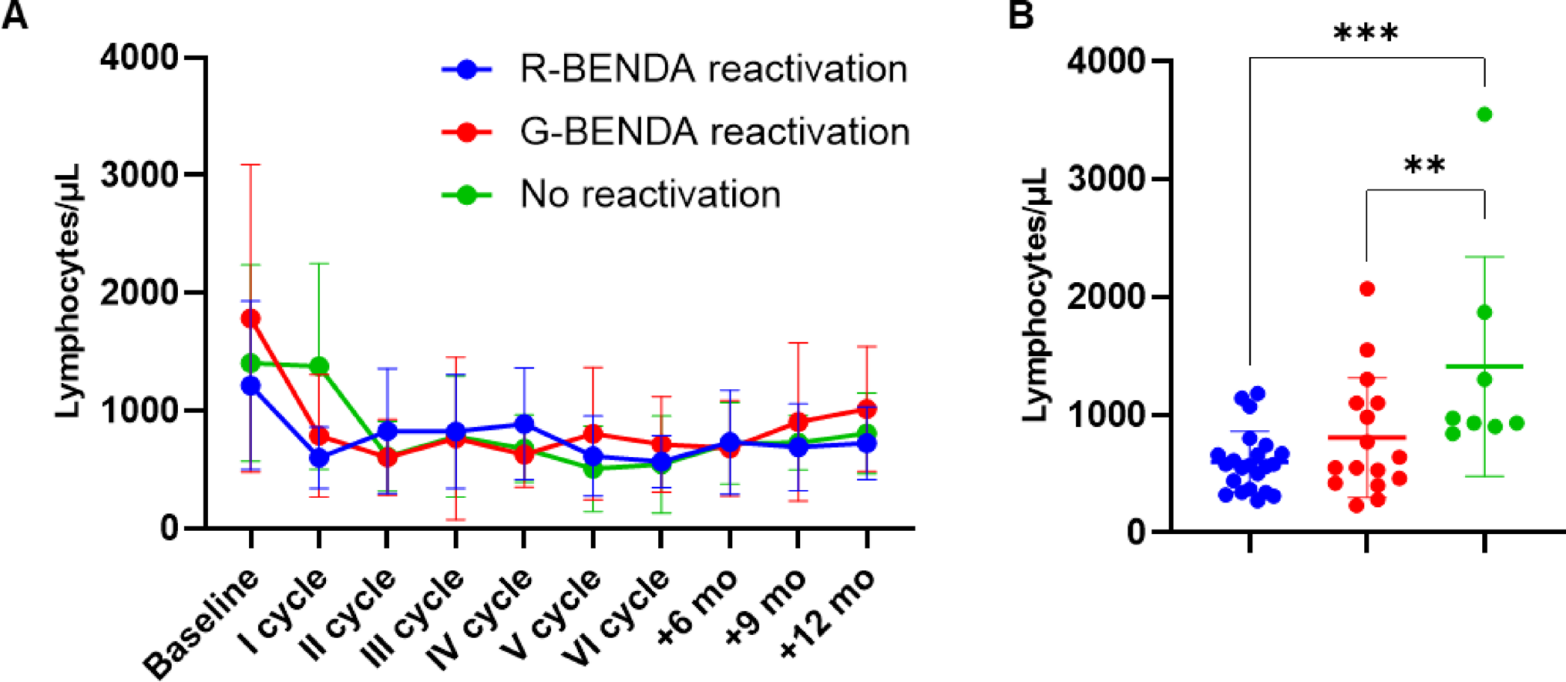
Immunological kinetics based on cytomegalovirus (CMV) reactivation. **A** Longitudinal analysis of lymphocyte counts stratified by reactivation status in patients treated with rituximab + bendamustine (R-BENDA), obinutuzumab + bendamustine (G-BENDA), and those without CMV reactivation regardless of the type of therapy. **B** Scatter plots of individual lymphocyte counts after the first cycle. Data are shown as mean ± SD. **P < 0.01; *** *P* < 0.001

To further delineate the relationship between immune reconstitution and viral reactivation, we stratified lymphocyte dynamics according to reactivation status within each treatment arm (Fig. 2). In all groups, lymphocyte count progressively declined and remained above normal ranges during follow-up. However, patients who experienced CMV reactivation, regardless of immunochemotherapy regimen, had a significantly lower lymphocyte count after the first cycle of therapy (mean ± SD, 603 ± 260 vs. 808 ± 508 vs. 1411 ± 929 cells/µL; *P* = 0.0019) (Fig. 2B).

## CMV reactivation incidence and clinical outcomes

**Fig. 3:**
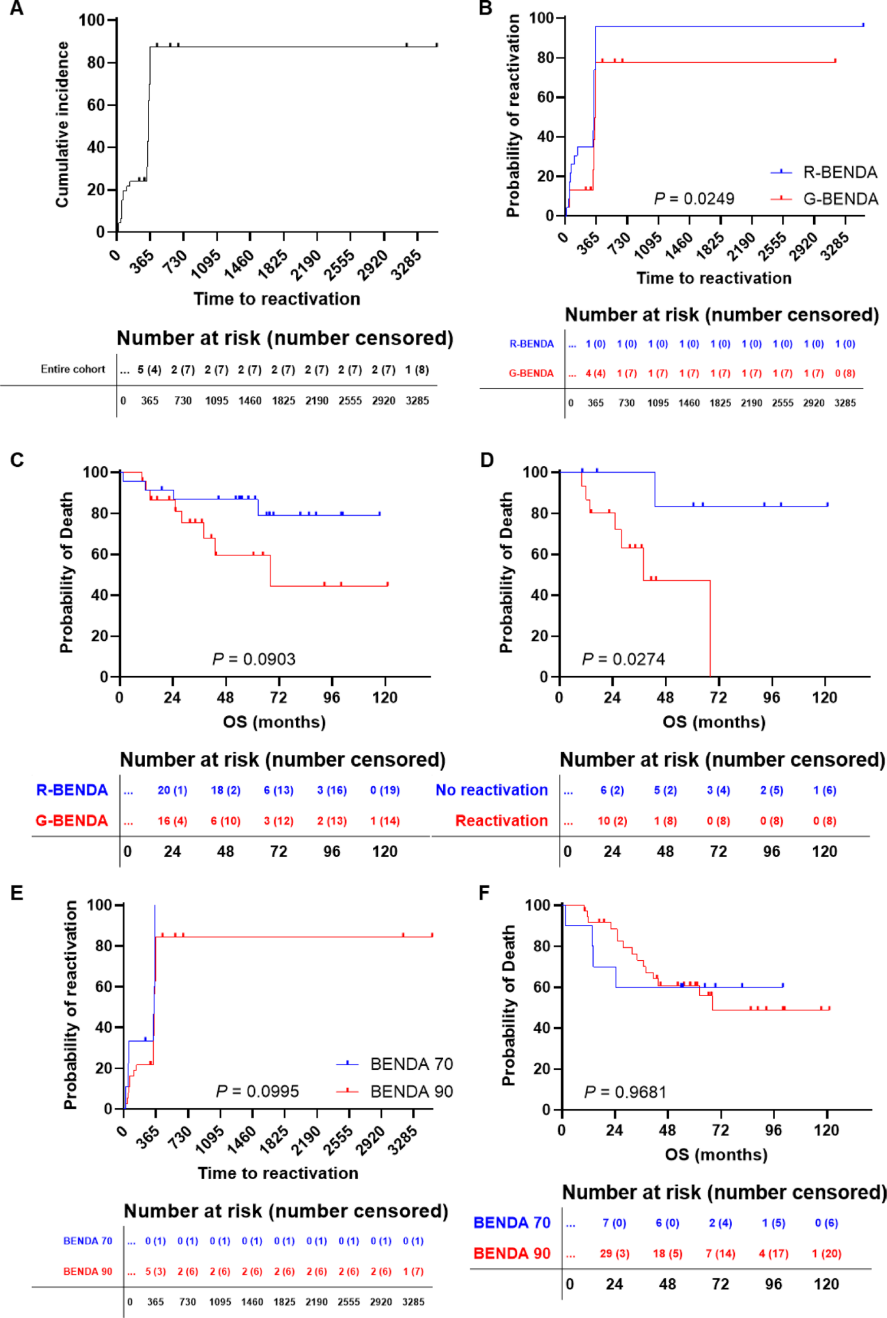
Cytomegalovirus (CMV) reactivation incidence and clinical outcomes. **A** Cumulative incidence of CMV reactivation in the entire cohort and in **B** patients receiving rituximab + bendamustine (R-BENDA) or obinutuzumab + bendamustine (G-BENDA). **C** Overall survival (OS) comparison between R-BENDA and G-BENDA treated patients, and **D** between G-BENDA treated subjects who experienced or not CMV reactivation. **E** Cumulative incidence of CMV reactivation and **F** OS in FL patients divided based on bendamustine dose: 70 mg/m^2^ (BENDA 70) or 90 mg/m^2^ (BENDA 90). Tables below the number of patients at risk and the number censored at each timepoint

In our FL cohort, the median time to CMV reactivation was 346 days (95% confidence interval [CI], 339–357 days) (Fig. 3A), slightly shorter in the R-BENDA group compared to G-BENDA (341 vs. 358 days, respectively; *P* = 0.0249) and a higher risk of reactivation (hazard ratio [HR], 2.232; 95% CI 1.107–4.5) (Fig. 3B). The 6-month incidence was 34.8% in the R-BENDA patients and 13% in the G-BENDA arm. Indeed, viral reactivation occurred in 96% of R-BENDA-treated patients (*N* = 22) and in 65% of G-BENDA (*N* = 15; relative risk, 1.467; *P* = 0.0220). Next, we investigated the impact of CMV reactivation on overall survival (OS), showing that G-BENDA-treated patients displayed a worse OS compared to R-BENDA group (2-year OS, 86.7% vs. 59.3%, R-BENDA vs. G-BENDA; *P* = 0.0903) (Fig. 3C). Subsequently, in each group, patients were divided based on the development of CMV reactivation, and OS was compared between populations only in the G-BENDA group, because almost the entire R-BENDA cohort experienced viral reactivation. As expected, G-BENDA-treated patients who developed CMV reactivation displayed a worse OS compared to those who did not experience viral reactivation (2-year OS, 47.3% vs. 83.3%, reactivation vs. no reactivation; HR, 5.245; 95% CI 1.202 to 22.88; *P* = 0.0274) (Fig. 3D). However, this difference represents a non-significant trend and needs confirmation in larger prospective multicenter studies. Of those with CMV reactivation, similar proportions of patients received anti-CMV therapies in both arms (*N* = 6 and *N* = 5, R-BENDA and G-BENDA arm; *P* = 0.7277). Two patients in the R-BENDA group required valganciclovir at 450 mg twice per day due to fever, while in the G-BENDA arm, one subject received valganciclovir at 450 mg four times daily and one patient switched from acyclovir to valganciclovir 1000 mg four times daily due to progressive increase of CMV viremia. In addition, one subject in the G-BENDA arm displayed two consecutive CMV reactivations treated with acyclovir at 800 mg daily. Mean treatment time was similar between groups (169 days [range, 42–381 days] vs. 129 days [17–218 days], R-BENDA vs. G-BENDA; *P* = 0.7846).

We further explored whether bendamustine dose influenced the risk and timing of viral reactivation, and patients were divided based on bendamustine dose administered: at 70 mg/m^2^ (BENDA 70) or 90 mg/m^2^ (BENDA 90) and clinical outcomes compared between groups. No statistically significant differences were observed in CMV reactivation incidence (median time to reactivation, 342 days vs. 346 days, BENDA 70 vs. BENDA 90; *P* = 0.0995) (Fig. 3E) and in OS (2-year OS, 60% vs. 60.8%; *P* = 0.9681) (Fig. 3F).

### Predictors of CMV reactivation

To identify independent predictors of CMV reactivation in FL patients treated with anti-CD20 moAb + bendamustine, a multivariate analysis was performed in the entire cohort, and in R-BENDA and G-BENDA cohorts (Table 2). No risk factors were identified in the entire population, while in the R-BENDA group, two clinical variables emerged as significant determinants of earlier reactivation: the presence of B symptoms associated with shorter time to reactivation (estimate, 2218; 95% CI 847.6–3588; *P* = 0.0088); and baseline SUV at staging PET scan, inversely correlated with time to reactivation (estimate, − 100.0; 95% CI − 180.1 to − 19.96; *P* = 0.0237). In the G-BENDA cohort, only B symptoms were associated with shorter time to reactivation (estimate, 1398; 95% CI 441.5–2355; *P* = 0.0086). Finally, we carried out a multivariate analysis to identify predictors of worse OS (Table 3). On the entire cohort, only the presence of B symptoms was associated with worse outcomes (estimate, 51.67; 95% CI 9.5–93.84; *P* = 0.0190), while no clinically significant associations were found in the R-BENDA group. Conversely, in the G-BENDA arm, platelet counts at baseline were associated (estimate, − 0.0003; 95% CI − 0.001 to − 0.000003; *P* = 0.0339). Although in the G-BENDA group there was a higher number of nodal involvements, this clinical feature did not influence clinical outcomes in both groups (*P* = 0.7174 and *P* = 0.6162, R-BENDA and G-BENDA).

**Table 2:** Multivariate analysis (dependent variable = time to reactivation)

Variable	Estimate	95% CI	|t|	*P* value
Entire cohort				
Intercept	492.8	− 866.9 to 1853	0.7615	0.4562
Age	− 0.493	− 9.270 to 8.285	0.1179	0.9074
SEX [F]	− 85.95	− 211.0 to 39.06	1.444	0.1658
Hb	− 13.80	− 56.66 to 29.07	0.6761	0.5076
PLT	0.001	− 9.012e−005 to 0.0022	1.938	0.0684
Symptoms [B]	132.0	− 111.7 to 375.7	1.138	0.2700
LDH	− 0.117	− 0.5651 to 0.3321	0.5456	0.5921
Albumin	− 35.51	− 180.5 to 109.4	0.5146	0.6131
No. of lymph nodes	− 4.448	− 23.21 to 14.32	0.4980	0.6245
SUV MAX	− 8.846	− 20.29 to 2.594	1.625	0.1216
Lymphocytes at baseline	0.0004	− 0.0577 to 0.0584	0.0132	0.9896
First line [R-BENDA]	− 26.53	− 144.1 to 91.00	0.4742	0.6411
Bendamustine dose	1.621	− 5.470 to 8.713	0.4804	0.6367
R-BENDA cohort				
Intercept	6785	− 2585 to 16,155	1.862	0.1217
Age	− 1.922	− 55.46 to 51.61	0.09229	0.9300
SEX [M]	678.3	− 222.3 to 1579	1.936	0.1106
Hb	− 228.7	− 528.6 to 71.21	1.960	0.1073
PLT	0.003	− 0.005 to 0.0119	1.027	0.3516
Symptoms [B]	2218	847.6 to 3588	4.161	0.0088
LDH	− 1.149	− 4.169 to 1.870	0.9785	0.3728
Albumin	− 846.1	− 2043 to 351.2	1.817	0.1290
No. of lymph nodes	9.237	− 127.1 to 145.5	0.1742	0.8685
SUV MAX	− 100.0	− 180.1 to − 19.96	3.211	0.0237
Lymphocytes at baseline	− 0.104	− 0.772 to 0.564	0.3994	0.7061
Bendamustine dose	6.999	− 40.00 to 54.00	0.3828	0.7176
G-BENDA cohort				
Intercept	− 5514	− 13,191 to 2163	1.600	0.1406
Age	27.52	− 19.34 to 74.39	1.309	0.2200
SEX [F]	440.9	− 253.5 to 1135	1.415	0.1875
Hb	− 28.64	− 264.0 to 206.8	0.271	0.7919
PLT	− 0.0016	− 0.0068 to 0.0036	0.682	0.5105
Symptoms [B]	1398	441.5 to 2355	3.256	0.0086
LDH	0.409	− 2.708 to 3.526	0.292	0.7760
Albumin	493.2	− 223.7 to 1210	1.533	0.1563
No. of lymph nodes	22.39	− 33.44 to 78.21	0.894	0.3926
SUV MAX	− 23.35	− 93.28 to 46.58	0.744	0.4740
Lymphocytes at baseline	0.009	− 0.293 to 0.3098	0.063	0.9511
Bendamustine dose	27.61	− 24.87 to 80.09	1.172	0.2683

**Table 3:** Multivariate analysis (dependent variable = OS)

Variable	Estimate	95% CI	|t|	*P* value
Entire cohort				
Intercept	− 198.1	− 484.3 to 88.15	1.448	0.1638
Age	0.859	− 0.9347 to 2.653	1.003	0.3287
SEX [F]	16.42	− 10.52 to 43.36	1.276	0.2175
Hb	7.399	− 1.469 to 16.27	1.746	0.0969
PLT	− 4.465e−005	− 0.0003 to 0.0002	0.349	0.7313
Symptoms [B]	51.67	9.500 to 93.84	2.565	0.0190
LDH	0.001	− 0.0937 to 0.095	0.015	0.9886
Albumin	8.889	− 21.53 to 39.31	0.612	0.5481
No. of lymph nodes	− 0.516	− 4.074 to 3.042	0.304	0.7648
SUV MAX	− 2.063	− 4.447 to 0.3213	1.811	0.0860
Lymphocytes at baseline	− 0.003	− 0.015 to 0.008	0.608	0.5505
Bendamustine dose	0.873	− 0.6024 to 2.348	1.238	0.2306
CMV reactivation [YES]	12.88	− 17.71 to 43.47	0.881	0.3891
R-BENDA cohort				
Intercept	60.32	− 512.0 to 632.6	0.293	0.7844
Age	0.6056	− 1.999 to 3.210	0.646	0.5537
SEX [M]	− 5.184	− 60.09 to 49.73	0.262	0.8062
Hb	2.633	− 17.52 to 22.78	0.363	0.7351
PLT	4.299e−005	− 0.0004 to 0.0005	0.273	0.7986
Symptoms [B]	105.8	− 21.03 to 232.6	2.316	0.0815
LDH	− 0.09659	− 0.2516 to 0.0584	1.730	0.1587
Albumin	− 28.97	− 104.9 to 46.98	1.059	0.3493
No. of lymph nodes	− 0.9488	− 7.729 to 5.832	0.389	0.7174
SUV MAX	− 3.645	− 9.869 to 2.579	1.626	0.1793
Lymphocytes at baseline	− 0.007927	− 0.0409 to 0.0251	0.666	0.5420
Bendamustine dose	1.493	− 0.8690 to 3.855	1.755	0.1541
CMV reactivation [NO]	− 149.5	− 320.4 to 21.45	2.428	0.0721
G-BENDA cohort				
Intercept	172.8	− 179.9 to 525.4	1.108	0.2965
Age	− 0.8153	− 3.057 to 1.427	0.823	0.4320
SEX [F]	4.417	− 27.43 to 36.27	0.314	0.7609
Hb	2.012	− 8.743 to 12.77	0.423	0.6821
PLT	− 0.0002650	− 0.0005 to − 2.520e−005	2.500	0.0339
Symptoms [B]	20.32	− 23.50 to 64.14	1.049	0.3215
LDH	− 0.03546	− 0.1782 to 0.1073	0.562	0.5878
Albumin	0.9973	− 31.95 to 33.95	0.069	0.9469
No. of lymph nodes	0.7267	− 2.440 to 3.893	0.519	0.6162
SUV MAX	− 0.4846	− 3.682 to 2.713	0.343	0.7396
Lymphocytes at baseline	0.003303	− 0.0111 to 0.0177	0.521	0.6152
Bendamustine dose	− 0.4176	− 2.913 to 2.077	0.379	0.7137
CMV reactivation [YES]	− 29.02	− 69.04 to 11.01	1.640	0.1354

## Discussion

In our exploratory single-center real-life two-arm study, we show that CMV reactivation is frequent during anti-CD20 + bendamustine induction for FL, with a higher incidence and rapid reactivation under R-BENDA regimen. These results are biologically coherent and clinically actionable, dovetailing with three data streams: (1) mechanistic differences between anti-CD20 antibodies used in clinical practice -rituximab versus obinutuzumab-; (2) bendamustine-driven T cell and humoral immunity suppression; and (3) growing real-world evidence linking bendamustine regimens to CMV disease.

Rituximab (type I) and obinutuzumab (type II, glycoengineered) vary in effector balance, as obinutuzumab enhances direct cell death and ADCC/ADCP, which might spare complement consumption and off-target humoral perturbations, explaining our observed early relative preservation of lymphocytes and CD4 + cells in G-BENDA after cycle I–II (Fujimura et al. 2021). However, under continuous treatment, obinutuzumab induces potent B cell depletion, while bendamustine synergistically causes T cell apoptosis, leading to lymphopenia, as already previously proposed in non-Hodgkin lymphomas treated with bendamustine-containing regimens (Weiner 2010; Tobinai et al. 2017; Herting et al. 2014; Lara et al. 2022). Indeed, bendamustine is consistently associated with prolonged lymphopenia, especially CD4 + depletion, delayed immune recovery (median 7–9 months), and a higher incidence of opportunistic infections (PJP, VZV, CMV) (Fung et al. 2019; Saito et al. 2015). Similar to that reported in literature, our longitudinal results were consistent with persistent and prolonged immunosuppression during and after bendamustine-based regimens (Lalic et al. 2022; Fung et al. 2019; Saito et al. 2015; Isono et al. 2016; Saburi et al. 2023; Pezzullo et al. 2021; Ito et al. 2024). Previous studies using data from the Japanese Adverse Drug Event Report have highlighted an increased risk of CMV reactivation in patients receiving anti-CD20 moAbs anti bendamustine, with reported odds ratios (ROR) of 16.3 [95%CI, 14.53–18.19] and 14.5 [95%CI, 10.62–19.69] for rituximab and obinutuzumab, respectively (Ito et al. 2024). Of note, although bendamustine-treated patients have a ROR of 22.2 [95%CI, 16.48–29.97], no additional risk of CMV reactivation has been observed when bendamustine is administered with rituximab (ROR, 17.9; 95%CI, 13.77–21.07) or obinutuzumab (ROR, 14.6; 95%CI, 10.28–20.78) (Ito et al. 2024). According to Japanese data, in our Italian case series of FL patients, we showed that the slightly higher risk of CMV reactivation in rituximab-treated patients could be linked to a more rapid lymphopenia and CD4 + T cell reduction, as G-BENDA regimen caused a deeper reduction in lymphocyte count after four cycles of therapy. Indeed, R-BENDA-treated patients displayed an early significant increased risk of CMV reactivation compared to G-BENDA subjects, with a 6-month incidence of 34.8% vs 13%, respectively. However, both regimens induced prolonged immunosuppression, and lymphocyte counts and Ig serum levels remained below normal ranges. Moreover, early immunosuppression after the first cycle of therapy could represent a predisposing condition for CMV reactivation, as FL patients in our cohort who did not experience viral reactivation had a higher lymphocyte count after the first cycle of bendamustine-based regimens, compared to those who had CMV reactivation regardless of the type of anti-CD20 moAb used. In addition, we clearly showed that bendamustine at any dosage did not increase the risk of CMV reactivation regardless of the type of anti-CD20 moAb used. Differences in CMV reactivation incidences could be principally linked to the different mechanisms of action of rituximab and obinutuzumab, and related B cell depletion. Indeed, chimeric antigen receptor (CAR) T cell therapies directed against CD19 or BCMA cause an increased incidence of CMV reactivation (19.6% or 27%, respectively), due to B lymphocyte ablation (Hayashino et al. 2025; Kampouri et al. 2024). Therefore, although T cells are essential in anti-CMV immune responses, B cells also play a crucial role, as reduced memory B cells are associated with low anti-CMV IgG titers and increased risk of reactivation after transplantation (Xia et al. 2017; Degli-Esposti and Hill 2022).

CMV reactivation develops early during bendamustine treatment, usually after the third cycle of therapy (Singhania et al. 2017; Isono et al. 2016; Hasegawa et al. 2015; Cona et al. 2019; Lim et al. 2012; Modvig et al. 2017; Hosoda et al. 2013; Yamasaki et al. 2017), as described in lymphoma patients with various histology and types of combinatorial regimens who showed a median time to reactivation within the first two months of therapy (Pezzullo et al. 2021). Potential predictors of CMV reactivation were: early CD4 + and absolute lymphocyte count after the first cycle of R-BENDA; high tumor burden; and the presence of B symptoms (Isono et al. 2016; Saburi et al. 2023; Pezzullo et al. 2021; Ito et al. 2024). In particular, B symptoms independently predicted earlier reactivation in both groups, plausibly reflecting systemic inflammation, cachexia, and cytokine milieus that degrade antiviral defenses. B symptoms and viral reactivations are not directly linked, while they share common contributing factors, such as disease- and drug-induced immunosuppression (Deans and Wigmore 2005). Although there were some discrepancies in patients’ characteristics between groups, such as a higher nodal involvement in G-BENDA group, no influences on clinical outcomes were observed by multivariate analysis for clinical and laboratory parameters associated with advanced stage disease (e.g., LDH levels, number of nodal involvements, and SUV max).

Given high CMV seroprevalence and incidence in FL patients, PCR-based surveillance during induction is reasonable for FL patients receiving anti-CD20 + bendamustine regimens, particularly those with B symptoms and/or high disease burden. The ECIL has provided consensus for hematology patients (most robustly in transplant recipients), emphasizing regular CMV viremia monitoring and pre-emptive therapy (Ljungman et al. 2019, 2025). At our Institution, CMV viremia is monthly measured at each cycle and weekly post-reactivation, which is pragmatic and concordant with expert guidance adapted to high-risk non-transplant settings (Pezzullo et al. 2021; Ljungman et al. 2019, 2025; Girmenia et al. 2025; Piñana et al. 2024). This approach allows prompt initiation of pre-emptive therapies, reducing the risk of CMV disease. For asymptomatic viremia with progressively increasing CMV-DNA, valganciclovir/ganciclovir remains the standard of care, maribavir is an option in refractory/resistant disease in transplant literature, while letermovir is established for prophylaxis in allogeneic hematopoietic stem cell transplantation, not routinely used in non-transplant lymphoma patients but of theoretically of interest for ultra-high-risk subsets (Ljungman et al. 2019, 2025; Girmenia et al. 2025; Piñana et al. 2024).

Rituximab-treated patients had the highest CMV reactivation incidence, while obinutuzumab-treated FL subjects who experienced viral reactivation displayed a shorter OS compared to those without reactivation. Of note, these worse clinical outcomes of our FL patients treated with G-BENDA could be also linked to the higher tumor burden at diagnosis compared to R-BENDA group, although no significant statistical associations were documented by multivariate analysis. Our results are in contrast with that reported in the GALLIUM and in the GOYA trials for HBV reactivation risk in FL and non-FL patients treated with G- or R-CHOP regimens (Kusumoto et al. 2019; Vitolo et al. 2017 Nov 1; Marcus et al. 2017). Indeed, a post-hoc analysis has revealed that HBV reactivation is more frequent in obinutuzumab-based regimen (13.2% incidence) compared to rituximab based (6.1%), although in the GOYA trial only diffuse large B cell lymphoma patients have been enrolled (Marcus et al. 2017). This discrepancy might be explained with the combinatorial use of bendamustine with anti-CD20 moAbs, as we have already shown that non-Hodgkin lymphoma patients treated with R-BENDA and R-BENDA + dexamethasone have the highest incidence of CMV reactivation compared to those subjects who receive bendamustine-free therapies (Pezzullo et al. 2021). Therefore, we added evidence on the synergistic immunosuppressive effects of bendamustine in combination with anti-CD20 moAbs, likely because of simultaneous T and B cell functional impairment and reduction (Pezzullo et al. 2021).

Our study has several limitations: (1) its retrospective single-center design; (2) limited sample size and deaths; (3) potential confounding by unmeasured variables (e.g., cumulative steroid exposure, antimicrobial prophylaxis, and CMV-specific T-cell immunity); (4) lack of randomization; (5) lack of long-term follow-up data, including progression and relapse rates; (6) lack of measurement of specific anti-CMV T cell immunity; and (7) the small number of subjects in each arm, that could increase the risk of type II errors (false negatives) due to biological variability, therefore, we could not exclude additional statistically significant differences between groups.

In conclusion, FL patients receiving anti-CD20 moAbs + bendamustine induction have increased risk of CMV reactivation, with a higher incidence during R-BENDA treatment, which causes a rapid lymphocyte and CD4 + T cell decline. Conversely, G-BENDA induces a progressive but deeper suppression with cumulative cycles, with late reactivations observed. B symptoms consistently predict earlier reactivation, and SUV at baseline could represent an additional predictor of CMV reactivation in R-BENDA-treated patients. Therefore, our data support routine CMV serology at baseline and PCR surveillance during immunochemotherapy, with pre-emptive antiviral strategies on rising viremia, and comprehensive infection prophylaxis tailored to risk. However, larger prospective randomized trials are required to test whether risk-adapted monitoring or targeted prophylaxis can reduce clinically significant CMV events without compromising anti-lymphoma efficacy, and to assess the real clinical impact of CMV reactivation and disease on outcomes.

## Data Availability

Data are available upon request by the Authors.
